# Polyphenols, Oxidative Stress, and Metabolic Syndrome

**DOI:** 10.1155/2020/7398453

**Published:** 2020-01-22

**Authors:** Chong Tian, Lei Hao, Weijie Yi, Shibin Ding, Fangyi Xu

**Affiliations:** ^1^School of Nursing, Tongji Medical College, Huazhong University of Science and Technology, Wuhan, China; ^2^Indiana University of Pennsylvania, USA; ^3^Binzhou Medical University, Shandong, China; ^4^Xinxiang Medical University, Henan, China; ^5^University of Louisville, Kentucky, USA

Metabolic syndrome (MetS), referring to a series of metabolic abnormalities, including central obesity, hyperglycemia, dyslipidemia, and hypertension, is common soil for many kinds of chronic diseases. Oxidative stress, which is commonly associated with MetS, has been proven to contribute to diabetes, atherogenesis, hypertension, and other cardiovascular diseases. Increasing evidence showed that polyphenols, a group of antioxidative phytochemicals, could protect cells against oxidative damage and, therefore, reduce the risk of associated diseases.

The objective of this special issue is to present how polyphenols protect against MetS and related disorders. Five among the many papers submitted were included after a thorough peer review process.

Nuts are polyphenol-rich but also hypercaloric food. L. Di Renzo et al. conducted a prospective pilot clinical trial and showed that extra calorie intake through hazelnuts leads to favorable blood lipid changes but not weight gain. Investigation of genomic response demonstrated that upregulation of genes implied in oxidative reactions and inflammation may be the mechanism underneath. This pilot study implied that nuts, although calorie dense, could be included in weight loss and anti-inflammatory diets.

Due to the wide-spanning and diverse distribution of polyphenols in foods, self-reported dietary intake suffers from errors. Meanwhile, validated *in vivo* biomarkers of polyphenols are still lacking. Precise assessment of polyphenol intake remains a challenge in epidemiological and clinical researches in this area. R. Yang et al. developed and validated a targeted metabolomics method by LC/MS/MS for the measurement of twenty-two polyphenol biomarkers in urine samples. The levels of the biomarkers were inversely associated with overweight and obesity. The research offered alternative polyphenol biomarkers and a complementary addition to self-reported survey for the evaluation of polyphenol intake.

Indoxyl Sulfate (IS), a uremic toxin derived from tryptophan metabolite by gut microbiota, is capable of exerting preoxidant effects on the cardiovascular system and accelerate vascular dysfunction. E. Getachew Assefa et al. investigated the protective effect of resveratrol against endothelial hyperpermeability induced by IS. The mechanism study showed that blockade of IS-aryl hydrocarbon receptor mediates Src activation and downregulates the activity of VE-cadherin and endothelial hyperpermeability.

Abnormal systemic oxidative stress, which is closely related to systemic inflammation, endothelial dysfunction, metabolic abnormality, and DNA damage, is proposed to be the most important pathophysiology of MetS. K. Liu et al. summarized evidences of the bioprotective effects of polyphenols against MetS and reviewed related antioxidative mechanisms, which involves oxygen scavenging, oxidoreductase enzyme system balancing, antioxidant responsive signaling pathway regulating, and mitochondrial function restoring effects. These data deepens our understanding of polyphenolic compounds and may help provide new insight in the development of future antioxidant therapeutics.

Intestinal health, especially the integrity of intestinal barrier, plays a crucial role in the whole organism. Y. Zhuang et al. demonstrated that resveratrol could directly protect IPEC-J2 cells against oxidative stress through the PI3K/Akt-mediated Nrf2 signaling pathway in an oxidative stress model induced by H_2_O_2_*in vitro*, which provided important evidence of a potential cytoprotective effect of resveratrol in the intestinal barrier.

All of these studies provide readers new insight regarding the evidences and mechanisms of polyphenols reducing oxidative stress-induced damage in the contexts of MetS. We are confident that these findings have contributed to researches concerning polyphenols, oxidative stress, and metabolic syndrome.

